# The Utilization of the SaLux19-Based Loop-Mediated Isothermal Amplification (LAMP) Assay for the Rapid and Sensitive Identification of Minute Amounts of a Biological Specimen

**DOI:** 10.3390/life14050579

**Published:** 2024-04-30

**Authors:** Ema Ruszova, Daniel Vanek, Walter Stühmer, Ziad Khaznadar, Nidhi Subhashini

**Affiliations:** 1 Bulovka University Hospital, 180 81 Prague, Czech Republic; 2 Forensic DNA Service, Budinova 2, 180 81 Prague, Czech Republic; 3 Institute for Environmental Sciences, Charles University, 110 00 Prague, Czech Republic; 4 Department of Forensic Medicine, Second Faculty of Medicine, Charles University, 110 00 Prague, Czech Republic; 5 Max Planck Institute of Multidisciplinary Sciences, Hermann-Rein-Str. 3, 37075 Göttingen, Germany; 6 Seratec GmbH, Ernst-Ruhstrat-Str. 5, 37079 Göttingen, Germany

**Keywords:** species identification, LAMP, point-of-care testing, SaLux19, *Sus scrofa* (wild boar/pig)

## Abstract

Our research has developed a highly sensitive and simple assay to detect small amounts of animal and human biological material in less than 40 min. The handheld SaLux19 device developed at the Max Planck Institute of Experimental Medicine in Göttingen, Germany, was used to validate our concept. The proposed system uses isothermal amplification of DNA in a rapid assay format. Our results show that the assay can detect *Sus scrofa* nucleic acids with very high sensitivity and specificity. This detection system has potential for forensic scenarios.

## 1. Introduction

Detecting minute amounts of biological samples and identifying the species is crucial for various reasons. In food safety, it ensures accurate labelling and detection of contaminants. In disease diagnosis, early detection of pathogens or genetic markers allows for timely intervention and treatment, potentially saving lives and preventing outbreaks. In forensic science, it aids in crime scene investigations, linking suspects to crime scenes or victims ultimately aiding in the pursuit of justice. Meeting precision in biological sample detection is critical, especially in scenarios where only trace amounts of material are available. This demand of precision and accuracy is facilitated by sensitive and specific detection methods like loop-mediated isothermal amplification (LAMP) [1]. LAMP offers several advantages, including high sensitivity and specificity, rapid results, and simplicity of operation [2]. These attributes make LAMP particularly well suited for applications requiring the detection of low-abundance targets or the amplification of DNA under challenging conditions, such as those encountered in forensic investigations or point-of-care diagnostics.

LAMP assays are highly specific (due to the large number of primer pairs), generate much more amplicon DNA (deoxyribonucleic acid) than conventional polymerase chain reaction (PCR), and can detect the amplification product by colorimetry or fluorescence. Fluorescent indicators (dyes) can be included in a LAMP reaction, which, as expected, provides greater sensitivity than colorimetric detection [3]. LAMP allows specific amplification of the target nucleic acids more efficiently than traditional PCR [4] and, most importantly, under isothermal conditions [5,6]. The simplicity of this screening method allows for fast and straightforward DNA analysis. Second, the nature of isothermal amplification massively reduces the complexity of the instrument that must be designed and built without the need for sequential thermocycling stages and the associated expensive and specialized thermocycling equipment, most often restricted to a dedicated laboratory [7].

Current methods based on DNA analysis, which determine the presence of species-specific biological materials have excellent sensitivity [8], but due to lack of on-site testing facilities or equipment, the duration of testing is significantly high, providing hinderance to prompt actions. This study is aimed to develop an in-house method that exploits a novel amplification technique called loop-mediated isothermal amplification (LAMP), particularly using the device SaLux19 (Max-Planck Innovation prototype, Göttingen, Germany), allowing fluorescent real-time control of amplification and focusing on DNA identification from *Sus scrofa* [9] biological fluid (venous blood) from forensically sized samples (~2 μL or less, diluted 100×, 1000×, at their appropriate concentration units within the range from ng to pg of DNA [10]). The species for this study was selected to be *Sus scrofa* as identifying pig species in food is crucial for several reasons. Firstly, pork consumption is forbidden in certain religions such as Islam and Judaism [11,12]. Hence accidental consumption of swine tissue can lead to religious concerns and dietary restrictions. Secondly, pork allergies are common, and consuming pig species can trigger allergic reactions in sensitive individuals [12,13]. The accurate labelling of food products helps prevent allergic reactions and ensures consumer safety. Last but not the least, mislabelling or adulteration of food products with cheaper substitutes can deceive consumers and compromise food quality and safety. Detecting pig species helps prevent food fraud and ensures authenticity and compliance with labelling regulations. Hence, our current study will provide assurance to consumers, regulatory authorities, and religious communities regarding food authenticity, safety, and observance of dietary restrictions.

## 2. Materials and Methods

### 2.1. SaLux19 Device

Principle of real-time SaLux19 point-of-care (POC) based testing

**Figure 1:**
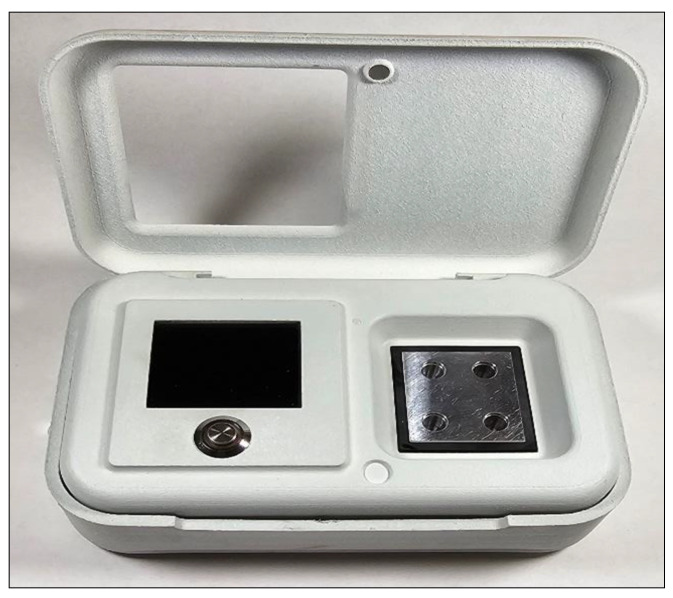
The SaLux19 device.

This pocket-size device has dimensions of 14.5 cm × 9.5 cm × 4 cm (L × W × H, Figure 1) and is equipped with a portable power adapter. By nature, the SaLux19 device can detect specific DNA or cDNA genetic codes. This point-of-care (POC) modality allows the determination of both, reverse-transcribed mRNA–based pathogens or DNA pathogens and all biological tissues containing DNA specific to the corresponding species and individuals. The specificity of detection is based on the match in the sequence of a piece of DNA with synthetic “baits” (primers) that bind to the target to be analysed. The SaLux19 device is set to a constant working temperature of 64 °C. The principle exploits the presence of DNA polymerase with strand-displacement activity, newly formed stem-loop structures, and a new self-primed 3′-end creation [7]. A fluorescent included in the reagents containing the primers binds to any newly formed double helix. The binding of the fluorescent dye causes a shift in the fluorescent colour of the dye. This shift in fluorescence is detected by a photodetector behind an optical filter selective for the shifted fluorescent, illuminated by a light-emitting diode excitation light filtering out the shorter emission light. Therefore, the photodetector only detects the shifted fluorescence, whose intensity is proportional to the amount of newly formed double helix. The method is known as loop-mediated isothermal amplification (LAMP) and is utilized by the SaLux19 device. During the LAMP reaction, the amount of shifted fluorescence increases exponentially with time until saturation is reached due to the limited reagent. In this sense, a chain reaction is initiated by the presence of a few molecules of the agent to be detected, and the increase in fluorescence correlates with the input amount of DNA and is analogous to the Ct values obtained by the thermocycling PCR equipment. The Salux display displays the increase in newly formed double helixes in real time. As in PCR, the equivalent Ct value is calculated based on the time needed to detect a significant rise in fluorescence. The maximum observation time necessary for the SaLux19 device to detect only a few molecules of the specific substance to be analysed is expected to be 40 min.

### 2.2. Sample Preparation

Fresh blood from *Sus scrofa domesticus* was collected directly into blood collection tubes with EDTA, VACUETTE^®^ during regular pig slaughter. The collection of blood was carried out by the veterinary stations’ staff where the animals were slaughtered. The scope of the examination was requested according to the indication at the place of origin of the pigs (according to the registration number of the farm), for diseases—brucellosis, Aujeszky’s disease and classical swine fever. The human DNA control (Thermo Fisher Scientific) was purchased directly as a commercial product. Similar to another product: PowerQuant^®^ Male gDNA Standard (Promega, Madison, WI, USA)—such controls are produced as a pool from multiple male donors.

### 2.3. DNA Extraction and Quantification

DNA extraction ensures the purity and integrity of the DNA template, which is crucial for accurate amplification. The DNA was extracted using the Quick-DNA Miniprep Kit (cat. no. D3024) from ZymoResearch, Irvine, CA, USA. The amount of nuclear-DNA (nDNA) was measured using a standard curve constructed by amplifying known amounts of human DNA control (cat. no. 431266) from Thermo Fisher Scientific, Waltham, MA, USA. The sample volume used for DNA extraction with the Quick-DNA Miniprep Kit was the same as described in the corresponding instructions—100 μL *Sus scrofa* whole blood and the elution volume was set to 50 μL. In the case of direct lysis, 10 μL of whole blood was lysed.

Absolute quantification of the nuclear-DNA of *Sus scrofa*

DNA quantification ensures that the correct amount of DNA is added to the LAMP reaction mixture, optimizing the amplification process. It helps achieve reliable and consistent results by standardizing the input DNA across different samples. The DNA was extracted from nonprecipitating *Sus scrofa* blood, and its quantity was measured using iQ Sybr Green Supermix qPCR (cat. no. 1708880, Bio-Rad, Hercules, CA, USA). The TATA-binding protein (TBP) gene was targeted for quantification, being a 2-allele single gene [14,15]. The TBP genes from various species were aligned in ClustalO (https://www.ebi.ac.uk/ (accessed on 11 September 2023)), and primers were designed in PrimerQuest^TM^ online Tool (Integrated DNA Technologies Inc., Coralville, Iowa, USA) based on their most homologous sequences. Absolute quantification of the nuclear DNA (nDNA) was performed by PCR of the human DNA control (ThermoFisher) in a series of dilutions. Primers for the “backbone” TBP gene were used (PCR output in Supplementary Figure S1). The DNA from *Sus scrofa* (100× diluted:brown colour) was simultaneously tested. In a second step, real-time PCR was performed to calculate the relative portion of mitochondrial DNA in the *Sus scrofa* sample (Supplementary Figure S2). This was carried out to fully characterize the *Sus scrofa* sample.

Each reaction had a volume of 20 µL and contained 1× Supermix, 10 μM forward and reverse primers (could be sent upon request), and 2 µL of unknown DNA. The TaqMan human control DNA was standard and ranged from 0.001 to 10 ng/µL. The samples were run in duplicates on a CFX Connect Real-Time PCR System (Bio-Rad, Hercules, CA, USA) at 95 °C for 3 min. Then, 40 cycles of 95 °C for 15 s and 58 °C for 45 s were performed. The CFX Manager Dx software v. 3.1 (Bio-Rad, Hercules, CA, USA) automatically determined the threshold cycle (Ct).

Relative quantification and determination of the mitochondrial-DNA to nuclear-DNA (mtDNA:nDNA) ratio

The mitochondrial-to-nuclear DNA ratio can provide insights into various biological processes, including cellular metabolism, mitochondrial function, and cellular stress responses. Changes in the mtDNA:nDNA ratio may indicate alterations in mitochondrial biogenesis, dynamics, or damage. Understanding this ratio is crucial for studying mitochondrial disorders, ageing, cancer, and other conditions in which mitochondrial dysfunction plays a role and hence determines whether the sampled animal was a diseased outlier or a healthy individual representing the normal population. Relative quantification of the mtDNA was performed using the same iQ SYBR Green Supermix (cat. No. 1708880, Bio-Rad, Hercules, CA, USA). The changes in the relative quantity of mtDNA to nDNA were determined as the ratio of the number of copies of the mitochondrial 16S gene to that of the TBP gene (nDNA) in the same tube. The adapted mathematical formula by Livak [16], 2 × 2^ΔCt(Ct(n) − Ct(mt))^, was used for analysis exploiting the difference between the ct values of the nuclear DNA (Ct(n)) and mitochondrial DNA (Ct(mt)). Amplification was carried out in duplicates for each DNA sample. The amplification primers used in the present study were the same as the ones published in the study of Boessenkool et al. [17], and the amplification temperature profile was the same as that used for absolute quantification. We used two dilutions of *Sus scrofa* DNA for the LAMP assay experiments. The first dilution was 100× and contained approximately 127 pg/μL of nDNA, with a ratio of mtDNA/nDNA roughly equal to 8.75. The second dilution was 1000× and contained approximately 13 pg/μL of nDNA. We performed direct lysis instead of the standard procedures as an alternative method. To achieve this, we utilized a DipStick DNA Extraction Kit (Bento Bioworks Ltd., London, UK) [18], which involves breaking open cells and releasing the DNA without additional purification steps. We found this alternative approach to be prompt and efficient for our needs.

### 2.4. Primers

(1) LAMP primers targeting the mitochondrial cytochrome c oxidase subunit I (COI) gene of *Sus scrofa* (accession number MN124249.1) were obtained from Jawla et al. 2021 [19] and synthesized by Eurofins Genomics Europe Shared Services GmbH (Ebersberg, Germany). The size of the LAMP product was forensically friendly at 226 base pairs (bp).
(2) LAMP primers targeting the human Y-amelogenin gene (AMEL-Y) (accession number NG_008011.2) were used for monitoring male DNA amplification using TaqMan™ Control Genomic DNA (cat. no. 4312660, Thermo Fisher Scientific, USA). The primers used were acquired from the published article of Nogami et al. 2008 [20] and synthesized by Eurofins Genomics Europe Shared Services GmbH (Ebersberg, Germany). The length of the amplification product (202 bp) was sufficient for detecting AMEL-Y in body fluids, as demonstrated in another study [21].

### 2.5. LAMP Assay

**Figure 2:**
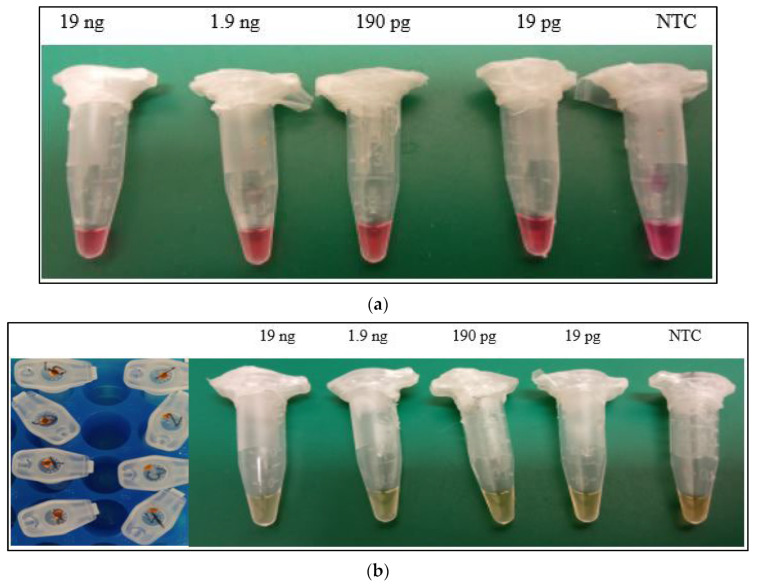

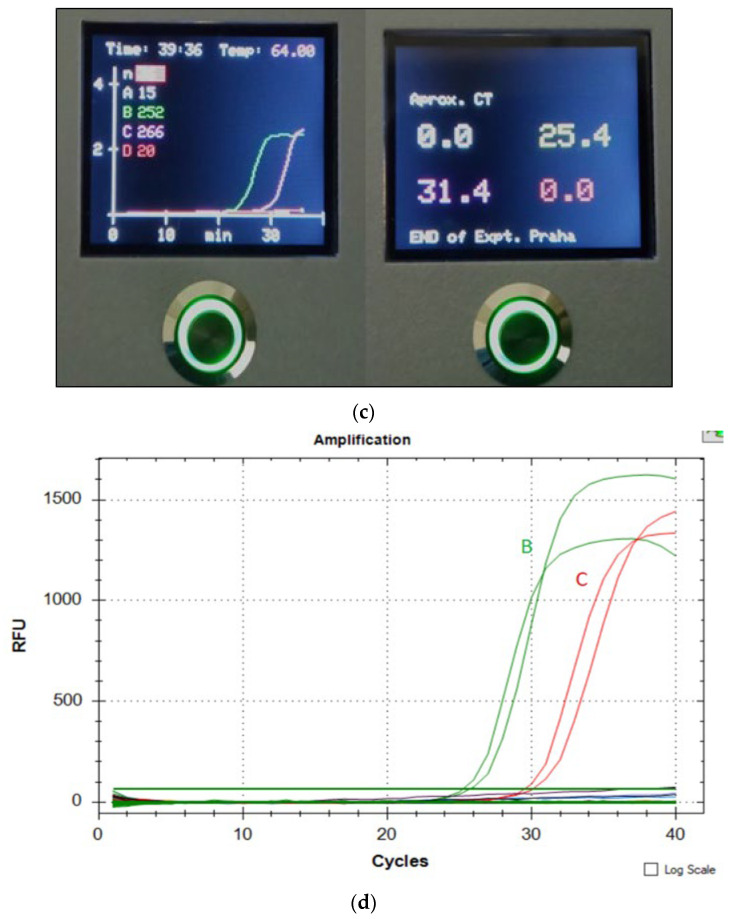
(**a**) LAMP assay for *Sus scrofa* detection using pH indicator phenol red. Results evaluated visually. NTC (no template control) included. (**b**) LAMP assay for detecting *Sus scrofa* using colorimetric changes with SYBR Green Gel Stain. Results evaluated visually. NTC (no template control) included. (**c**) The in-built display of SaLux19 device demonstrates real-time amplification. (**d**) Real-time SALUX19 assay, 100× diluted ≈ 127 pg/μL of nDNA (position B), 1000× diluted ≈ 13 pg/μL of nDNA (position C). Two other positions (A and D) were used as NTCs—no template controls. The fluorescent dye SYBR Green Gel Stain was used. An analogous test with the same concentrations of *Sus scrofa* DNA was performed using a CFX Connect Real-Time PCR system (Bio-Rad, Hercules, CA, USA).

Preliminary tests for the identification of *Sus scrofa* DNA were performed precisely according to the New England Biolabs (NEB, Ipswich, MA, USA) web protocols with visual control of colour pH changes [22] (Figure 2a) or the typical LAMP protocol [23], where the intercalating dye SYBR Green Gel Stain (cat.no. S7563, Thermo Fisher Scientific, USA), at a dilution of 1:10, was loaded into the inner part of the tube lid (Figure 2b). The reactions were carried out in 0.5 mL reaction microtubes within a thermoblock (Eppendorf, Germany).

Own real-time LAMP assay optimization was performed on a real-time PCR system (CFX Connect PCR system, Bio-Rad, Hercules, CA, USA) and the SaLux19 device (MPI-SERATEC, Göttingen, Germany). The protocol used was identical to the protocol used on the NEB website [22] and included the same concentration of primer mixture and the addition of 10,000× diluted SYBR Green Gel Stain (cat. no. S7563, Thermo Fisher Scientific, USA) or recommended fluorescent LAMP dye (cat. no. B1700S, NEB, Ipswich, MA, USA). LAMP was performed according to the written instructions on the NEB website [22,23,24] (Figure 2c,d).

### 2.6. Assay Validation

Here, we explored a method based on isothermal amplification to rapidly identify DNA originating from *Sus scrofa* on the SaLux19 device. Assay validation reactions were prepared in a final reaction volume of 25 μL with nuclease-free water and incubated at 64 °C for 40 min on a CFX Connect PCR system (Bio-Rad, Hercules, CA, USA) or SaLux19 (MPI-SERATEC, Germany). The Nerbe-Plus 0.1 mL microtubes (cat. no. 04-032-0350 Nerbe-Plus GmbH & Co. KG, Winsen, Germany) proved helpful for both real-time devices.

DNA samples from both eukaryotic and prokaryotic species, e.g., *Panthera tigris*, *Panthera leo*, *Panthera pardus*, *Caracal serval*, and *Bos taurus* “Salers bull”, the latter mentioned as a representative of phylogenetically related Artiodactyla, further *Homo sapiens* and the ZymoBIOMICS^®^ Microbial Community Standard (ZymoResearch, Irvine, CA, USA), were cross-tested for assay specificity. Prokaryotic DNA was isolated from 75 µL of the Microbial DNA Standard, ZymoResearch, using the same column extraction techniques (according to the manufacturer’s written instructions) [25]. The total DNA concentration was measured using only the Qubit fluorometric assay and diluted to a final concentration of ≈5 ng/μL, which was used once for the real-time assay. The nuclear DNA content of the other big cat samples was measured using the 4N6QuantALU quantification kit (Forensic DNA Service, Prague, Czech Republic). Their yield was in the span of dozens to hundreds of pg/μL.: (*P. leo* ≈ 230 pg/µL of nDNA, *P. tigris* ≈ 570 pg/µL of nDNA, *P. pardus* ≈ 30 pg/µL of nDNA). The DNA extracted from *P. serval* and *B. taurus* were measured only fluorometrically using Qubit (624 pg/µL of total DNA, respective 9.8 ng/µL). Each DNA sample was used in different ranges: 100× diluted DNA (*P. leo*, *P. tigris*, *P. serval*, *Bos taurus*), 50× diluted DNA (*P. pardus*).

**Figure 3:**
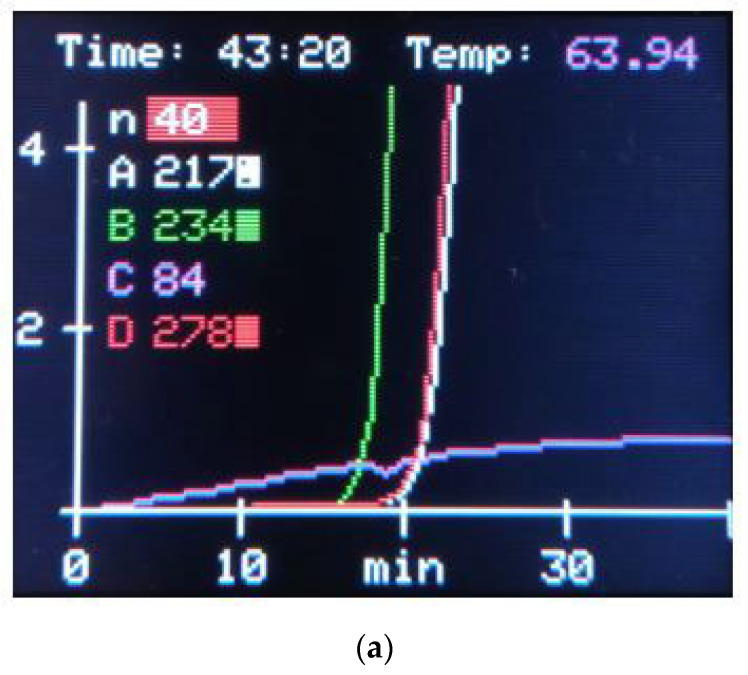

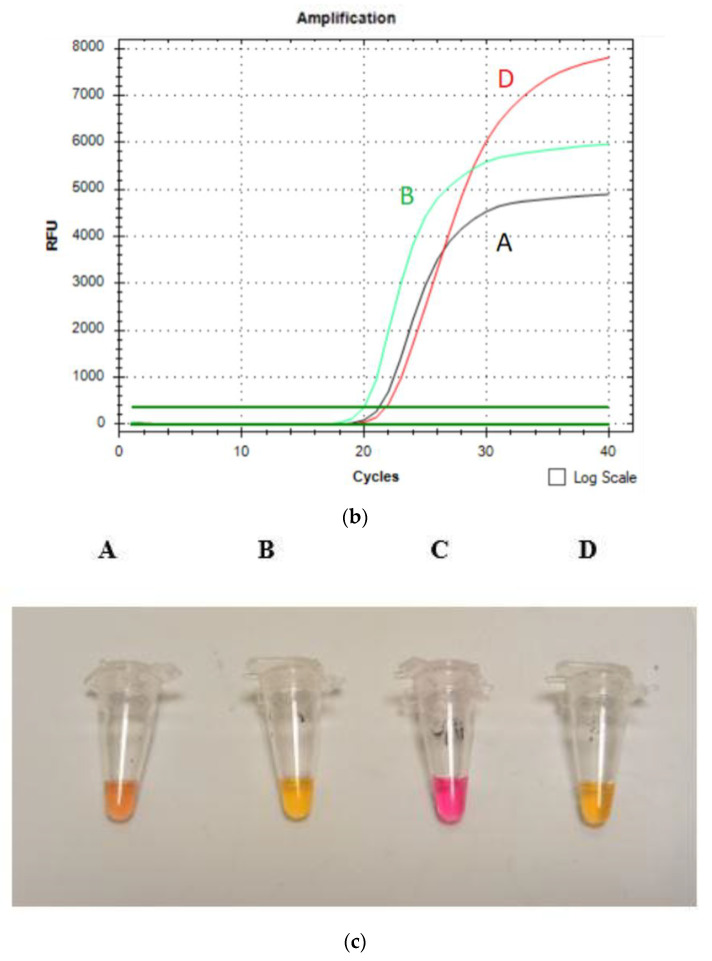
(**a**) The standard LAMP assay run on SaLux19 *Sus scrofa* cells. Sample A (Position A): 10 μL of *Sus scrofa* whole nonprecipitating blood lysed in direct DipStick extraction buffer. Positive control (Position B): *Sus scrofa* DNA at a concentration of 127 pg/μL nDNA. Human male DNA control (Position D): 30 copies of the AMEL-Y gene for checking amplification. No template control (NTC, Position C): Control sample without any template DNA. (**b**) An analogous test with the same concentrations of *Sus scrofa* DNA was performed using a CFX Connect Real-Time PCR system (Bio-Rad, Hercules, CA, USA). Sample A (Position A): 10 μL of *Sus scrofa* whole nonprecipitating blood lysed in direct DipStick extraction buffer. Positive control (Position B): *Sus scrofa* DNA at a concentration of 127 pg/μL nDNA. Human male DNA control (Position D): 30 copies of the AMEL-Y gene for checking amplification. No template control (NTC, Position C): Control sample without any template DNA. (**c**) The colorimetric detection assay was performed in parallel as quality control of the amplification experiments.

A specificity test revealed no amplification signal for any of the examined species. When the SYBR Green Gel Stain was replaced with a fluorescent dye for the LAMP assays, there was a significant increase in *Y*-axis-based relative fluorescent units (RFUs), as shown in Figure 3a,b.

Positive control for amplification

Human male TaqMan™ Control Genomic DNA (cat. no. 431266; Thermo Fisher Scientific, USA) was used as a positive control for amplification. We prepared a dilution series according to the Thermo Fisher application notes [26]. Once defrosted, the standard was diluted into the needed aliquots and stored at –20 °C to prevent repeated freeze–thaw cycles. When assessing sensitivity, successfull amplification of the human AMEL-Y target was achieved by the repeated use of at least 10 copies of this DNA control. Human DNA control (ThermoFisher) was diluted exactly according to the Table 1 resulting in 303 to 3 in copy numbers.

Test arrangement

The standardized SaLux19 test protocol aiming for species identification includes both LAMP diagnostic reagents, with primer cocktails in all four positions and various samples for parallel amplification. One of the four wells contains a test sample, while two other wells are incorporated with a suitable target that serves as a control for a successful LAMP reaction (positive control). For this purpose, human male DNA focused on the Y-amelogenin gene was selected. Finally, one device position was occupied with no template control (NTC), allowing for control for the absence of foreign DNA (negative control).

## 3. Results

The final, standardized run on SaLux19 included 10 μL of *Sus scrofa* whole nonprecipitating blood lysed in direct DipStick extraction buffer (position A), *Sus scrofa* DNA at a concentration of 127 pg/μL nDNA as a positive control (position B), human male DNA at 30 copies of the AMEL-Y gene for checking out of own amplification (position D), and no template control (NTC, position C) as shown in Figure 3a. An analogous test with the same concentrations of *Sus scrofa* DNA was performed using a CFX Connect Real-Time PCR system (Bio-Rad, Hercules, CA, USA), Figure 3b. In addition to the possibility of real-time run monitoring, the addition of fluorescent dye directly to the colorimetric master mixes allows us to assess the results with the naked eye, as shown in Figure 3c.

As shown is Figure 3a, sample A containing the *Sus scrofa* whole nonprecipitating blood lysed in direct DipStick extraction buffer resulted in a relative fluorescence unit (RFU) value of 2175 ± 5 after 40 cycles; this indicates successful amplification of *Sus scrofa* DNA from the lysed blood sample. An RFU value of 2345 ± 5 was observed in Sample B with *Sus scrofa* DNA isolated from nonprecipitating blood. This indicates successful amplification of *Sus scrofa* DNA that serves as positive control, highlighting the functionality of the designed primers. Sample C, the no template control (NTC), had an RFU value of 84 after 40 cycles. This suggests the presence of background noise or nonspecific dye intercalation, possibly due to high concentration of oligos present in master mix. Sample D containing human male DNA resulted in an RFU value of 2785 ± 5; this indicates successful amplification of the human DNA target from the second positive control, verifying the functionality of other assay compartments (master mixes). Notably, significant differences in RFU among the four samples were observed after just 25 cycles, emphasizing the early detection capability of the assay. We performed analogous experiments with two systems in order to compare the amplification efficiency and the suitability of each fluorescent dye. The resulting Ct values were similar in both systems.

Overall, the results suggest successful amplification of the target DNA in samples A, B, and D, while sample C (NTC) shows some level of nonspecific amplification or background noise. The addition of fluorescent dye to the colorimetric master mixes allows for easy assessment of the results with the naked eye, facilitating result interpretation and quality control. Performing colorimetric detection in parallel to PCR offers a simple, cost-effective, and accessible approach for real-time monitoring and confirmation of DNA amplification assays, making it valuable for species identification.

## 4. Discussion

We report the successful design, optimization, preliminary testing, and validation of a portable and rapid DNA analysis system for identifying *Sus scrofa* body fluids using the SaLux19 device. Our LAMP-based point-of-demand (POD) testing system comes with fluorescent LAMP assay reagents. It is pocket sized and provides quick results. We are confident in the sensitivity of the LAMP analysis system, as our data indicate its effectiveness. We conducted successful specificity tests on a real-time CFX Connect PCR System (Bio-Rad, Hercules, CA, USA), confirming the reliability of the LAMP method, in this case the SaLux 19 device. The LAMP assay excluded eight prokaryotes (*Pseudomonas aeruginosa*, *Escherichia coli*, *Salmonella enterica*, *Lactobacillus fermentum*, *Enterococcus faecalis*, *Staphylococcus aureus*, *Listeria monocytogenes*, and *Bacillus subtilis*) and eight eukaryotic species (members of the big cat family and *Bovidae* mentioned in the assay validation section, *Homo sapiens,* and two yeasts: *Saccharomyces cerevisiae* and *Cryptococcus neoformans*).

Our system offers two key benefits: real-time reaction monitoring and the possibility to perform double evaluation. This feature enables quantitative fluorescent and colorimetric detection based on the colour change of the test tubes visible to the naked eye. The dual evaluation is facilitated by adding the fluorescent dye to the colorimetric master mix and transferring reaction mixtures from 0.5 mL thermoblock tubes to thin-walled 0.1 mL tubes, which permits a more efficient heat transfer and start-up in quantitative PCR or LAMP devices. A less time consuming procedure was achieved when blood was directly lysed with sodium-hydroxide-based DipStick buffer. We are confident that our system provides an efficient, reliable, and safe method for identifying DNA from *Sus scrofa* body fluids. The need for the rapid detection of biological analytes at their points of care (POC) led to the development of portable instruments enabling either nucleic acid amplification tests or antigen detection. LAMP assays, when coupled with user-friendly instruments, can deliver results in tens of minutes (SMART-LAMP [27], DNAiTECH Gen2 [28], GENIE II (OptiGene Ltd., Horsham, UK)) [29]. The SaLux19 instrument provides the added benefits of portability and the option of recharging via a USB adapter or even a small power bank.

Since the quality and integrity of the sample can affect the accuracy of species detection, it is important to consider the sizes of amplified targets for processed meat products. Degraded or contaminated samples may yield unreliable results due to potential DNA degradation during food processing which can adversely impact the amplification of longer amplicons. Several studies have reported that small amplicons with fragments less than 150 bp are recommended due to their increased sensitivity in limit of detection (LOD) in qPCR assays compared to longer fragments exceeding 200 bp [30]. On the other hand, several studies have demonstrated that the LAMP assay has the same detection capability as qPCR [31,32,33,34].

Although the presented research pipeline examined whole nonprecipitating blood, it remains to be elucidated how it would work on heat-processed meat products. Nonetheless, this methodology represents a rational, simple, robust, and low-cost approach for identifying *Sus scrofa* body fluids. It is also worth noting that species detection methods may be specific to certain target species and may not be suitable for identifying a wide range of species, limiting their applicability in diverse scenarios.

## 5. Conclusions

The development and validation of a rapid and portable DNA analysis system utilizing loop-mediated amplification (LAMP) on the SaLux19 device exhibit a significant leap forward in wildlife species identification and point-of-demand diagnostics. This study underlines the feasibility and effectiveness of employing LAMP technology for the specific and sensitive detection of *Sus scrofa* DNA, addressing critical needs for swift and accurate species identification, especially in contexts where pork consumption may be restricted or pose allergenic risks.

The SaLux19 device, characterized by its compact dimensions and real-time monitoring capabilities, presents a practical solution for on-site DNA analysis. Leveraging the isothermal amplification mechanism of LAMP, this device obviates the requirement for complex thermocycling equipment, thereby democratizing DNA analysis beyond specialized laboratory settings. Additionally, the incorporation of fluorescent LAMP assay reagents enables both real-time monitoring and dual evaluation, augmenting the system’s adaptability and reliability.

A promising strength of this system lies in its specificity, demonstrated through rigorous specificity testing encompassing various eukaryotic and prokaryotic species. The LAMP assay exhibited high specificity, excluding amplification signals from nontarget species, which is indispensable in forensic applications mandating precise species identification. Moreover, the LAMP assay showcased remarkable sensitivity, detecting *Sus scrofa* DNA even from minute samples at dilutions as low as 1000×. This capability to detect DNA from trace amounts of biological material underscores its utility in forensic investigations in which evidence may be scant. The integration of direct lysis as an alternative sample preparation method further streamlines the workflow, offering a swift and efficient approach for DNA extraction without the need for additional purification steps. This simplification of sample processing contributes to the system’s rapid turnaround time, vital in forensic contexts where prompt analysis is imperative.

Furthermore, the compatibility of the LAMP assay with both visual colorimetric detection and fluorescent real-time monitoring enhances result interpretation flexibility. Colorimetric detection facilitates rapid on-site screening through visual inspection, while fluorescent real-time monitoring enables quantitative analysis, strengthening the system’s utility in diverse research and clinical applications.

Despite the encouraging outcomes, further investigations are warranted to assess the system’s performance on processed meat products and to broaden its applicability to other species. These new questions hold promise for advancing rapid DNA analysis technologies, thereby propelling advancements in forensic science and public health. The SaLux19 device, with its compact design, real-time monitoring capabilities, and high specificity and sensitivity, emerges as a versatile and reliable tool for on-site DNA analysis, with potential applications spanning forensic investigations, food safety, and disease diagnosis. This study lays a robust foundation for future advancements in the realm of rapid DNA analysis, poised to make substantial contributions to forensic science and public health initiatives.

## Figures and Tables

**Table 1:** Dilution strategy for human male genomic DNA and final copy number achieved.

Dilution	Source of Genomic DNA	Initial Concentration (pg/µL)	Volume DNA (µL)	Volume Diluent (µL)	Final Concentration (pg/µL)	Resulting Copy No. of Human DNA
1.	Stock	10,000	10	90	1000	303
2.	Dilution 1	1000	10	90	100	30.3
3.	Dilution 2	100	10	90	10	3

## Data Availability

The original contributions presented in the study are included in the article/Supplementary Materials, further inquiries can be directed to the corresponding author/s.

## Supplementary Materials

The following supporting information can be downloaded at: https://www.mdpi.com/article/10.3390/life14050579/s1, Figure S1: Examined sample of Sus scrofa DNA (100× diluted: brown colour) shown in (a); Calibration curve—amplified TBP (TATA binding protein) gene target, male Human DNA Standard (green—10 ng/μL, 1 ng/μL, 100 pg/μL) shown in (b); Figure S2: (a) Amplification of 16S gene (orange), TBP gene (blue) of Sus scrofa sample (100× diluted) allowing relative quantification in the same run as its absolute quantification. (b) Calibration curve generated with the male human DNA control (ThermoFisher, green—10 ng/μL, 1 ng/μL, 100 pg/μL) and TBP primers. (c) High resolution melting profiles of 16S gene, TBP (TATA binding protein) gene and the male human DNA control; Figure S3: The test of LAMP assay specificity; (a) Amplification of positive control Sus scrofa DNA; (b) A qPCR plate sheet containing names and Ct values of various samples when amplified with same primers.
