# Supplementary material for: The Utilization of the SaLux19-Based Loop-Mediated Isothermal Amplification (LAMP) Assay for the Rapid and Sensitive Identification of Minute Amounts of a Biological Specimen
Source: Life (Basel). 2024 Apr 30;14(5):579. doi: 10.3390/life14050579 (PMC11122329; doi:10.3390/life14050579)
Supplement: Supplementary file 1 [file life-14-00579-s001.zip › life-2916057-supplementary.pdf]

SUPPLEMENTARY FIGURES

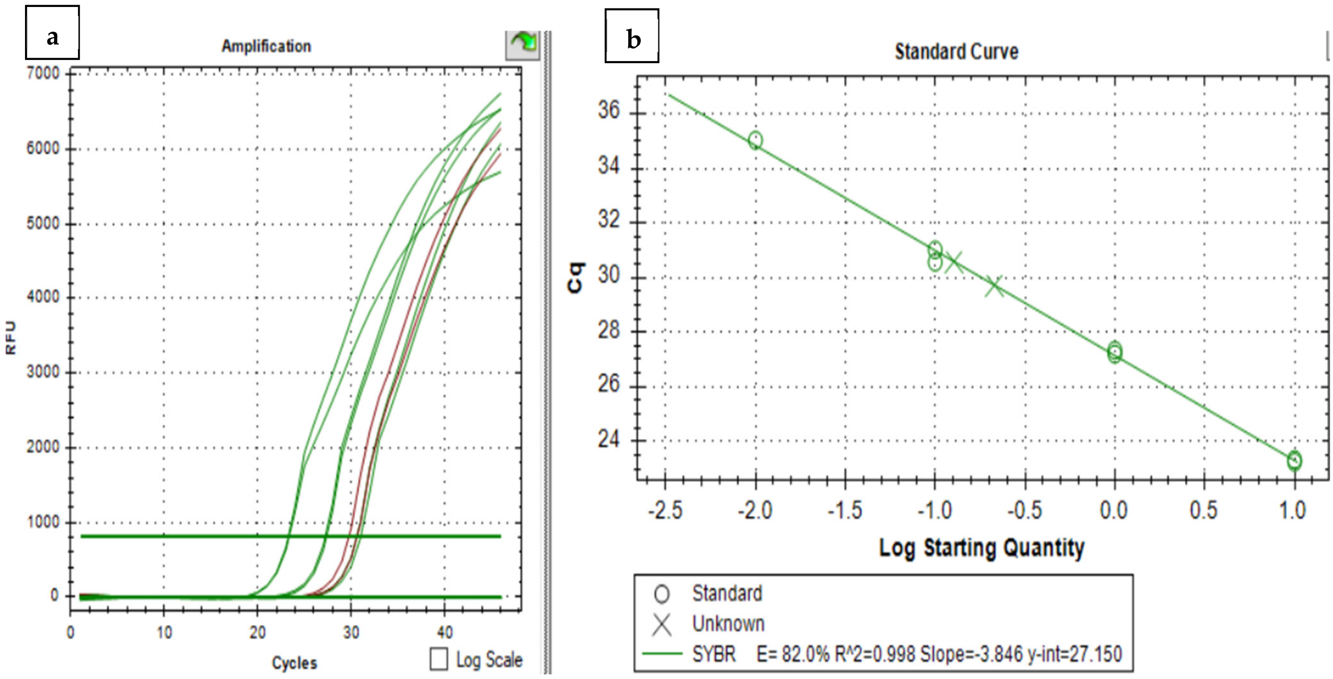

Supplementary Figure S1. Examined sample of *Sus scrofa* DNA (100xdiluted-brown color) shown in (a); Calibration curve—amplified TBP (TATA binding protein) gene target, male Human DNA Standard (green—10 ng/μL, 1 ng/μL, 100 pg/μL) shown in (b).

SUPPLEMENTARY FIGURES

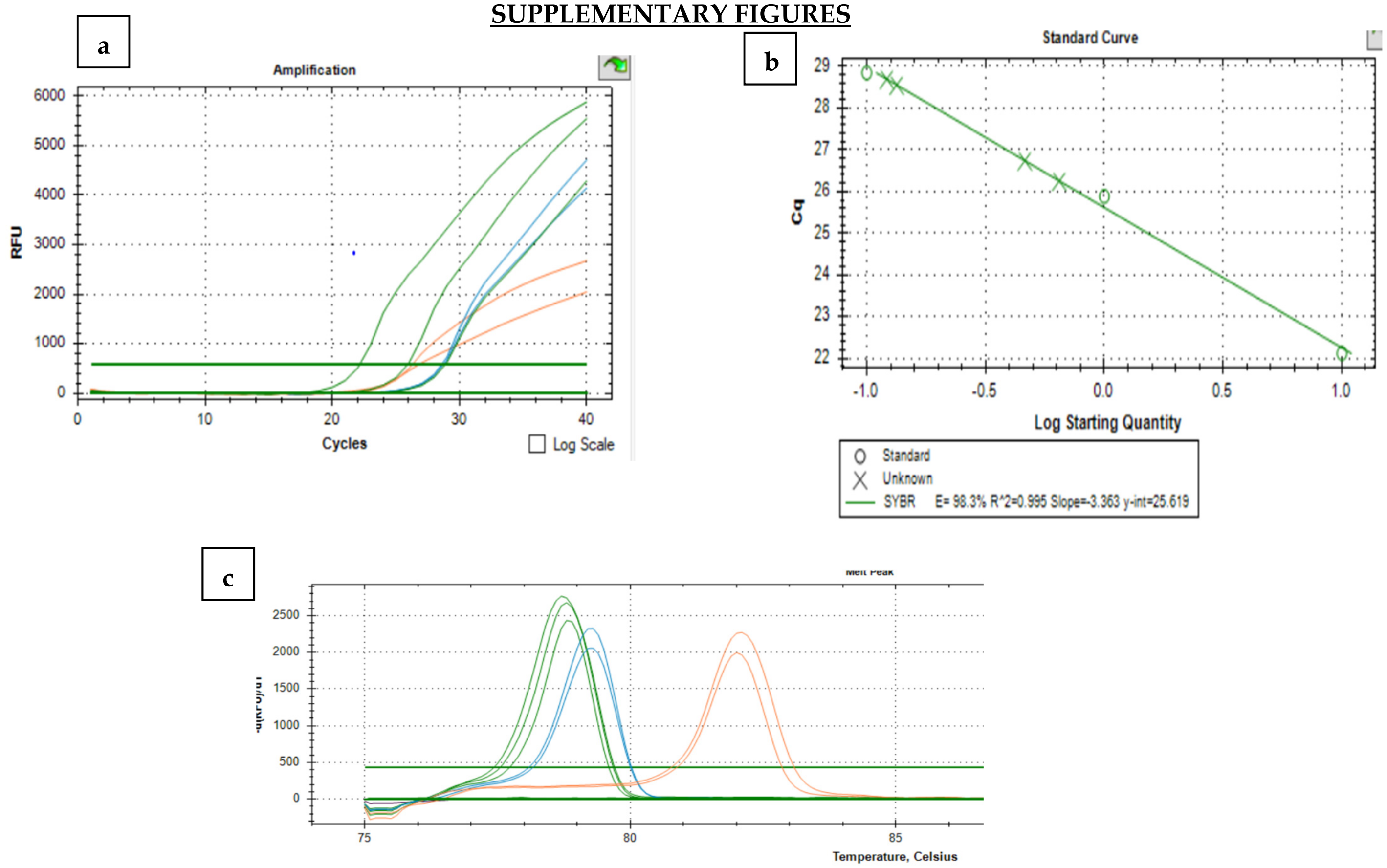

Supplementary Figure S2. (a) Amplification of 16S gene (orange), TBP gene (blue) of *Sus scrofa* sample (100× diluted) allowing relative quantification in the same run as its absolute quantification. (b) Calibration curve generated with the male human DNA control (ThermoFisher, green—10 ng/µL, 1 ng/µL, 100 pg/µL) and TBP primers. (c) High resolution melting profiles of 16S gene, TBP (TATA binding protein) gene and the male human DNA control.

SUPPLEMENTARY FIGURES

a

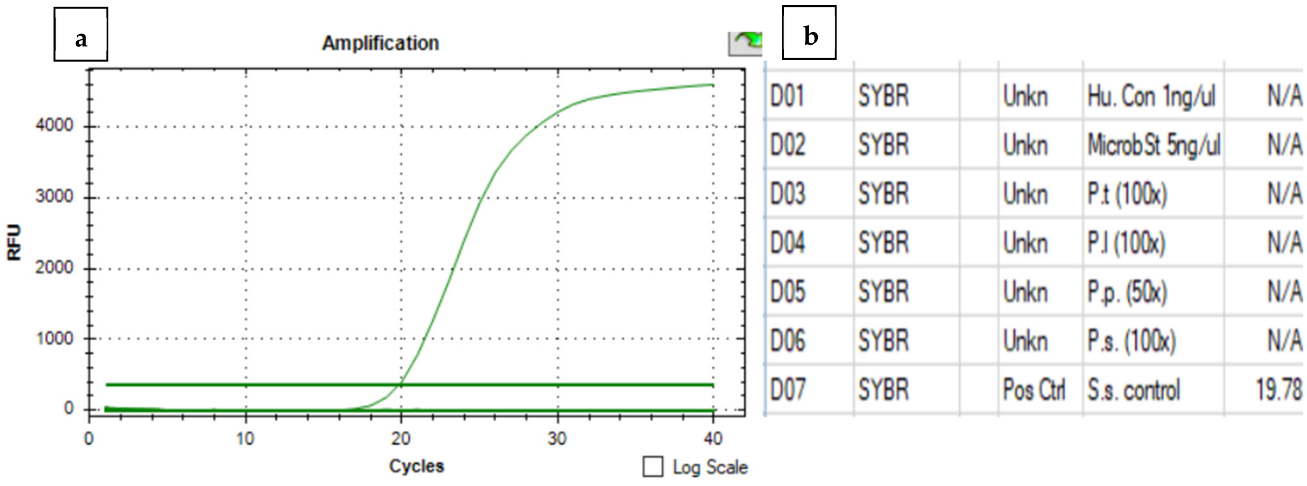

b

D01	SYBR	Unkn	Hu. Con 1ng/ul	N/A
D02	SYBR	Unkn	MicrobSt 5ng/ul	N/A
D03	SYBR	Unkn	P.t (100x)	N/A
D04	SYBR	Unkn	P.l (100x)	N/A
D05	SYBR	Unkn	P.p. (50x)	N/A
D06	SYBR	Unkn	P.s. (100x)	N/A
D07	SYBR	Pos Ctrl	S.s. control	19.78

Supplementary Figure S3. The test of LAMP assay specificity; (a) Amplification of positive control *Sus scrofa* DNA; (b) A qPCR plate sheet containing names and Ct values of various samples when amplified with same primers.
